# Integrative Genomic and Transcriptomic Analysis of Primary Malignant Gliomas Revealed Different Patterns Between Grades and Somatic Mutations Related to Glioblastoma Prognosis

**DOI:** 10.3389/fmolb.2022.873042

**Published:** 2022-07-05

**Authors:** Huawei Jin, Zhenhua Yu, Tian Tian, Guoping Shen, Weian Chen, Miao Fan, Qun He, Dandan Dai, Xuan Zhang, Dawei Liu

**Affiliations:** ^1^ Department of Neurosurgery, the First Affiliated Hospital of Sun Yat-sen University, Guangzhou, China; ^2^ Department of Nuclear Medicine, the First Affiliated Hospital of Sun Yat-sen University, Guangzhou, China; ^3^ Department of Radiation Oncology, the First Affiliated Hospital of Sun Yat-sen University, Guangzhou, China; ^4^ Department of Radiology, the First Affiliated Hospital of Sun Yat-sen University, Guangzhou, China; ^5^ GenomiCare Biotechnology (Shanghai) Co. Ltd, Shanghai, China; ^6^ Department of Pathology, the First Affiliated Hospital of Sun Yat-sen University, Guangzhou, China

**Keywords:** glioblastoma, genomic DNA, transcriptomic, somatic mutation, TME

## Abstract

**Background:** As reflected in the WHO classification of glioma since 2020, genomic information has been an important criterion in addition to histology for glioma classification. There is a significant intergrade difference as well as intragrade difference of survival probability among glioma patients. Except the molecular criteria used in the WHO classification, few studies have explored other genomic factors that may be underlying these survival differences, especially in Chinese populations. Here, we used integrative genomic approaches to characterize a Chinese glioma cohort to search for potential prognostic biomarkers.

**Methods:** We recruited 46 Chinese patients with primary malignant glioma. All the patients were analyzed with whole-exome sequencing (WES) and 27 of them were analyzed with RNA-seq. We compared the molecular features between patients in different WHO grades. We classified the glioblastoma (GBM) patients into two groups (good vs poor survival) using six-month progression-free survival (PFS6) status and compared the genomic profiles between the two groups.

**Results:** We found grade II and grade III patients cluster together (LGG) and they are different from GBM in unsupervised clustering analysis with RNA-seq data. Gene set enrichment analysis (GSEA) comparing GBM and the LGG group suggested that GBM had upregulation of multiple pathways related to genome integrity and immune cell infiltration. Further comparison of somatic mutations between the two groups revealed *TOPAZ1* as a novel mutation associated with GBM and prevalence of CNV in multiple genes in GBM. Comparison between PFS6 good and poor GBM patients revealed six genes (*TRIML2, ROCK1, PKD1, OBSCN, HECTD4, and ADCY7*) were significantly mutated and two genes (*NTRK1* and *B2M*) had more CNV alterations in the poor prognosis group.

**Conclusion:** Taken together, our molecular data revealed that GBM patient showed distinct characteristics related to individual gene, chromosome integrity, and infiltrating immune cells compared to LGG (grade II/III) patients. We also identified few novel genes with SNV or CNV, which might be the potential markers for clinical outcome of GBM.

## Introduction

Glioma is a common primary brain tumor, accounting for a quarter of all brain tumors (Mesfin and Al-Dhahir, 2021). It includes a very diverse group of tumors in the terms of histology, pathology, and clinical presentations. The World Health Organization (WHO) classification system had grouped gliomas into four grades considering mainly histology and malignancy, but added molecular feature as an important criterion in the recent revision of the classification system (Louis et al., 2007, Louis et al., 2016). Grade I gliomas are often benign and curable. Grade II (diffuse) and grade III (anaplastic) gliomas (LGG) are more aggressive and they can evolve into the highest grade gliomas, grade IV (glioblastoma, GBM) (Ohgaki and Kleihues, 2007). Grade II and III gliomas are often grouped together as low grade gliomas (LGG), and grade IV gliomas are often grouped together as high grade gliomas (HGG) in clinical practice because of the contrast between LGG and HGG, and resemblance within LGG itself or HGG itself in terms of treatment strategy and prognosis (Forst et al., 2014; Oberheim Bush and Chang, 2016). Nevertheless, GBM always flag extra attention from doctors and patients because they are extremely malignant and therefore occupy an independent domain in the clinical world. (Stupp et al., 2014). Standard treatment of malignant gliomas involves surgical resection followed by radiotherapy and temozolomide (TMZ)-based chemotherapy, sometimes referred as the Stupp protocol (Stupp et al., 2014). The protocol can prolong the survival of patients, especially for grade II or III gliomas. However, the prognosis of GBM is still poor, giving a 5-year survival rate of only 5% (Ostrom et al., 2018). Usually, GBM recurs within a year and there is no good therapy for those recurrent GBM patients (Gallego, 2015).

In the WHO classification of glioma published in 2016 and 2021, genetic characteristics are integrated with histopathological features for patient grading (Louis et al., 2016; 2021). According to previous studies, genomic information has shown marvelous correlation with prognosis. For example, *IDH1* mutation and 1p/19q codeletion define a favorable outcome, which often occur in grade II and III group (Yan et al., 2009; Boots-Sprenger et al., 2013). In *IDH1*-wildetype subgroup, patients tend to carry *EGFR* amplification, chromosome 7 gain and 10 loss, and have poor prognosis, and are classified as HGG (Stichel et al., 2018; Louis et al., 2021). Up to date, researches based on TCGA have revealed many molecular features of glioma, including mutations, CNVs, DNA methylation, and gene expression (Ceccarelli et al., 2016). However, few studies have integrated genomic and transcriptomic data with clinical survivability in Chinese population to explore the difference between grades. Considering the genetic heterogeneity of different human lineages and the impact of genetic factors on the prognosis of malignant glioma, it is necessary to further investigate the genetic characteristics of malignant glioma in Chinese population.

Here, we assembled a dataset comprising whole-exome sequencing (WES), RNA-seq, and clinical data from 46 patients with primary malignant glioma in Chinese population. We first did unsupervised clustering of patient RNA-seq data and used the result to divide patients into a GBM group and a LGG group. We then compared the groups according to their molecular features including somatic mutations, cellular pathways, and immuno-phenotypes. Additionally, we further divided the GBM patients into two subgroups by using their six-month progression-free survival time (PFS6) (Ballman et al., 2007) and compared their genomic landscape to search for prognostic markers.

## Materials and Methods

### Patients and Specimens

Fresh frozen or formalin-fixed paraffin-embedded (FFPE) tumor tissue specimens and matched peripheral blood from 46 primary glioma patients were retrospectively collected from the first affiliated hospital, Sun Yat-Sen University. Based on the histopathological diagnoses, following the 2016 WHO guidelines, the tumors were classified as grade II to IV gliomas. Most patients received chemotherapy after the first surgery as per the standard treatment protocol for primary glioma. All the specimens were profiled by WES and 27 of them were also analyzed by RNA-seq to give a transcriptomic profile. This study is approved by the institutional review board of the First Affiliated Hospital of Sun Yat-sen University. All the patients signed informed consent.

### WES and Data Processing

DNA was extracted from the paraffin-embedded (FFPE) tissue with MagMAX FFPE DNA/RNA Ultra kit (cat# A31881, ThermoFisher), or from the snap-frozen tissue and peripheral whole-blood with Maxwell RSC blood DNA kit (cat# AS1400, Promega). Matched peripheral whole blood was used as the normal (germline) control in filtering to call somatic mutations. Purified DNA was sheared and hybridized to probes from Agilent SureSelect XT Human All Exon V7 kit (cat# 5,991–9,039, Agilent). After exome enrichment, the captured DNA was amplified, end-repaired, and attached to adapters and barcode using SureSelect XT HS and Low Input Library Preparation Kit for ILM (PrePCR) kit (cat# G9704, Agilent). The prepared sequencing libraries were normalized and sequenced on Illumina NovaSeq-6000 Sequencing System to generate paired-end (150 × 150 bp) reads. The generated reads were preprocessed by removing the low-quality reads and then aligned to NCBI human genome reference assembly hg19 using the Burrows-Wheeler Aligner (BWA) alignment algorithm.

The further process was carried out using the Genome Analysis Toolkit (GATK, version 3.5) with its MuTect/ANNOVAR/dbNSFP31 and VarscanIndel add-on software to determine single-nucleotide polymorphism (SNP) and Indel, respectively. Copy number variation (CNV) was determined using CNVnator software (Abyzov et al., 2011). The called mutations were annotated using the Variant Effect Predictor (VEP) package (hg19 version) (McLaren et al., 2016). Specific features of somatic mutations were visualized using the R package Maftools (version 2.6.05) (Mayakonda et al., 2018). The extracted DNA (200 ng) was modified with bisulfite using the EZ-96 DNA Methylation-Lightning™ MagPrep (Zymo Research, Irvine, CA, United States ) according to the manufacturer’s instructions. The MGMT primer sequences were 5- GATATGTTGGGATAG TT-3 (sense) and 5-CCC​AAA​CAC​TCA​CCA​AAT-3 (antisense). The qPCR assays were performed using an ABI Prism 7900HT Sequence Detection System.

### RNA-Seq and Data Processing

RNA was extracted using the MagMAX FFPE DNA/RNA Ultra kit from the FFPE samples. Reverse-transcription and cDNA synthesis were done using the NEBNext RNA First-Strand Synthesis Module (cat# E7525S, NEB) and NEBNext Ultra II nondirectional RNA Second Strand Synthesis Module (cat# E6111S, NEB), respectively. RNA-seq libraries were prepared using the SureSelect XT HS and Low Input Library Preparation Kit for ILM (Pre PCR) (cat# G9704, Agilent), then sequenced on Illumina NovaSeq-6000 Sequencing System to generate the paired-end reads (150 × 150 bp). The obtained reads were mapped to the hg19 reference genome by using STAR (version 020201) and assembled using StringTie2 (version 1.3.5) with default parameters (Kovaka et al., 2019).

Further analysis of RNA-seq included unsupervised clustering and the normalized data were used as the input of the Gene Set Enrichment Analysis (GSEA) and single-sample GSEA (ssGSEA) (Mootha et al., 2003; Subramanian et al., 2005). The ssGSEA procedure was used to calculate the enrichment scores of gene sets (including KEGG and Immune sets) in each sample. The function gsva from the R package GSVA was used to do the ssGSEA analysis. The function GSEA from the python package gseapy was used to do the GSEA analysis. For the different enrichment gene sets was downloaded from the GSEA official website and was used as the KEGG set. A student t-test was used to filter significant differentially infiltrated immune cells. Heatmaps of differentially expressed gene sets were visualized with an R package.

### Statistical Analysis

All statistical analyses were performed using R (https://cran.r-project.org) or SPSS 25 for Windows (SPSS Inc., Chicago, IL, United States ). Differences in the distribution of somatic mutations, mutational signatures, and clinical characteristics between patient subgroups were evaluated by the Fisher’s exact test and Mann–Whitney *U* test for categorical and continuous parameters, respectively, and events of *p* < 0.05 were considered statistically significant.

The WES (*n* = 46) and RNA-seq (*n* = 27) data of each patient were submitted to the European Genome-phenome Archive (EGA) database with study ID: EGAS00001005583.

## Results

### Patient Characteristics

A total of 46 primary glioma patients were included in this study. The clinical characteristics of the patients were summarized in Table 1. The median age at the first onset was 41 (range from 12 to 85). This cohort consisted of 17 grade II, five grade III, and 24 grade IV glioma patients. Treatment before and after surgery included chemotherapy and/or radiotherapy or observation only. The majority of patients (38/46, 82.6%) received chemotherapy after the operation. All the patients were analyzed by WES. At least half of the patients were analyzed by RNA-seq in each grade, being 10, 3, and 14 patients for grade II, grade III, and GBM, respectively.

**TABLE 1:** Patient distribution with different grades and clinical features.

Features	Grade II (*n*=17)	Grade III (*n*=6)	Grade IV (*n*=23)
Sex			
Male	13	4	11
Female	4	2	12
Age			
Median (range)	34 (19–50)	52 (33–67)	52 (12–85)
Treatment			
Before surgery			
Chemotherapy	0	1	0
Radiotherapy	0	1	1
After surgery			
Chemotherapy	13	6	19
Radiotherapy	2	1	5
Molecular			
MGMT promoter			
Methylated	12	3	14
Unmethylated	5	3	7
Unknown	0	0	2
*IDH* status			
Wildtype	4	2	22
Mutant	13	4	1
Analytical platform			
WES	17	6	23
RNAseq	10	3	14

### Clustering Analysis

**FIGURE 1:**
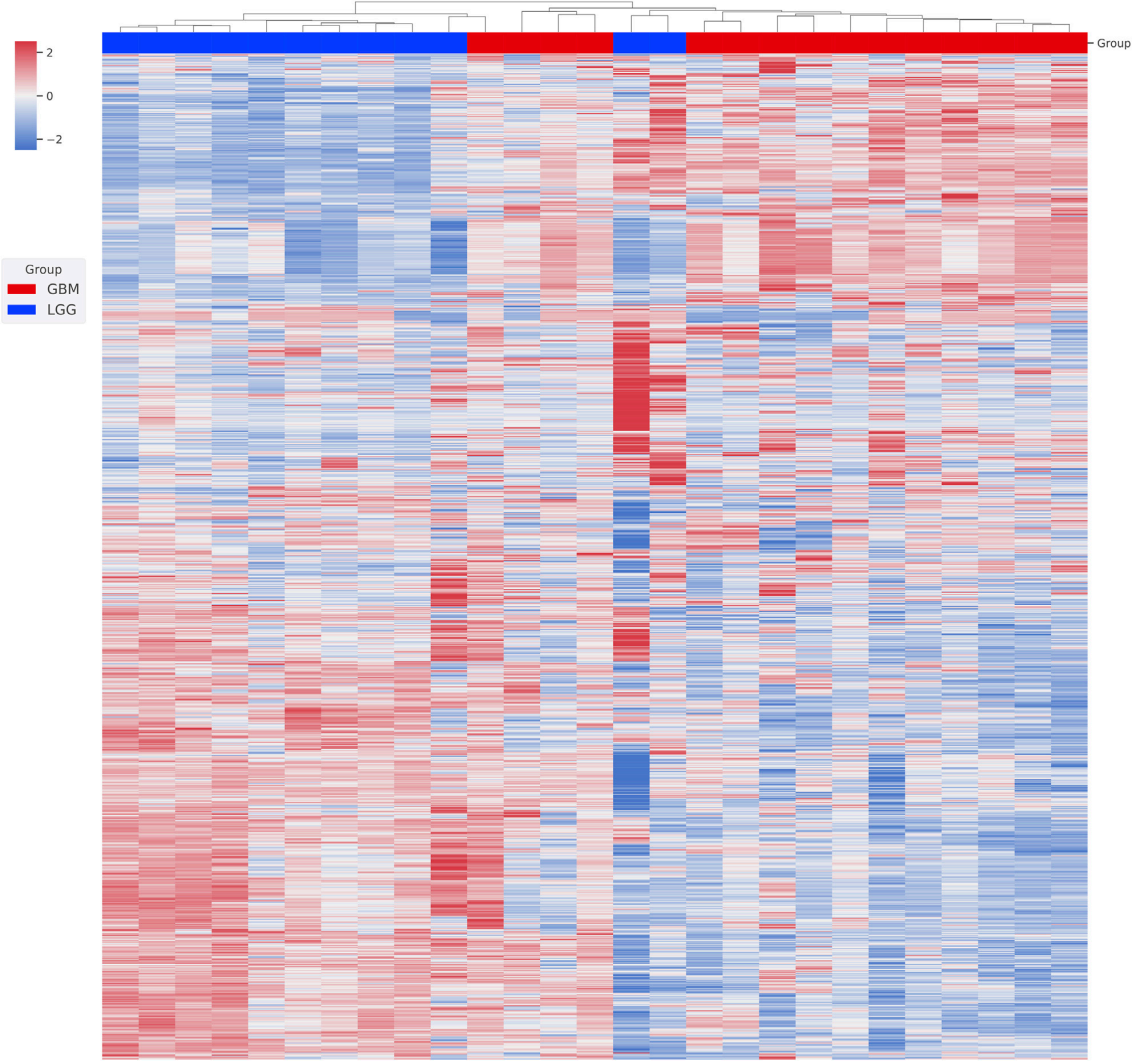
Unsupervised clustering of patient gene expression profiles. The transcriptome of each patient was first obtained from RNA-seq analysis. The top 2,000 genes with differential expression were then used in hierarchical clustering. Relative gene expression levels (from +2 to -2) are indicated by red and blue color shades. Patients’ WHO classifications at diagnosis are color coded.

We first performed unsupervised clustering on the available RNA-seq data to assess how the patients were similar or dissimilar to each other in their gene expression profiles (Figure 1). We found two major clusters. All 3 grade III patients and 8 out of 10 grade II patients together formed the first cluster. All the GBM patients and the other two grade II patients formed the second cluster. Grade II and grade III gliomas are grouped to LGG, and two group patients share many common features in clinical treatment consideration, prognosis, and also molecular characterization. Based on our clustering result, we also believe that grade II and grade III glioma patients share significant gene expression profiles and GBM is a distinct entity largely different from grade II and grade III gliomas. We, therefore, merged grade II and grade III patients together to form an LGG group and did a comparative analysis between them and GBM patients.

### RNA-Seq Revealed Differential Pathways and Immune Cells Between Grade II/III and GBM Groups

**FIGURE 2:**
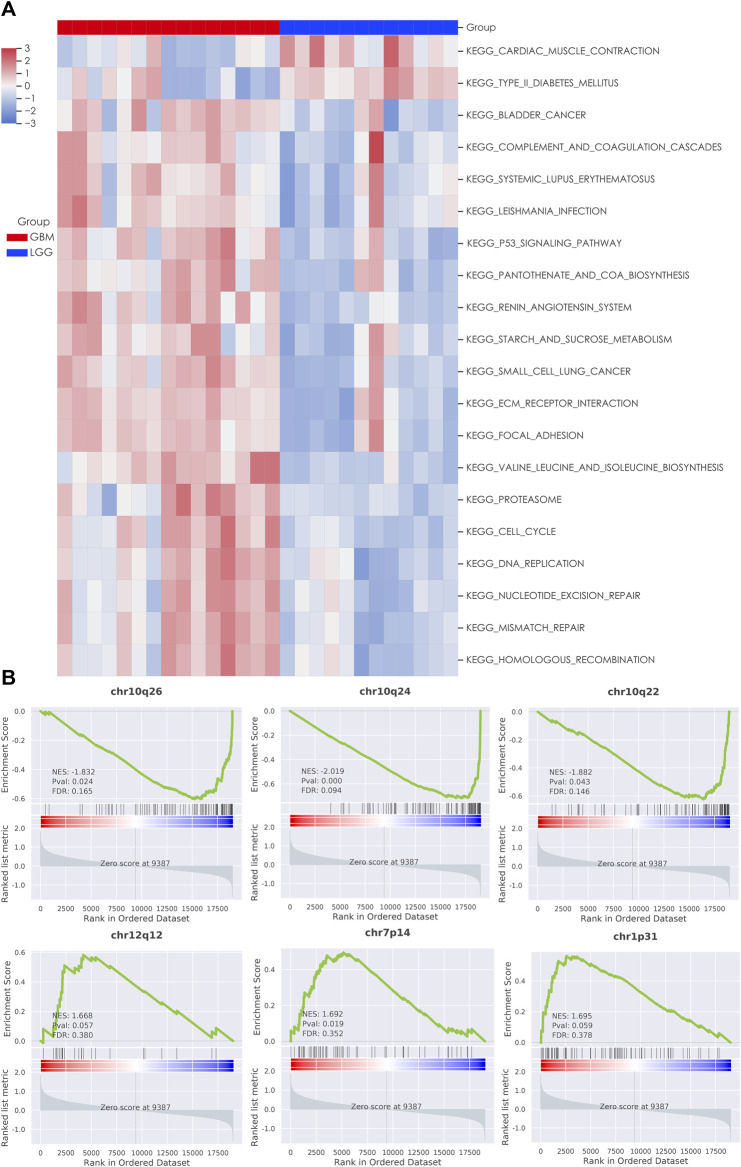
Gene set enrichment analysis (GSEA) of LGG and GBM transcriptomes. **(A)** Heatmap of significant KEGG pathways in patients; **(B)** enrichment plot of chromosomal locations with the most significant EnrichmentScores (ES).

**FIGURE 3:**
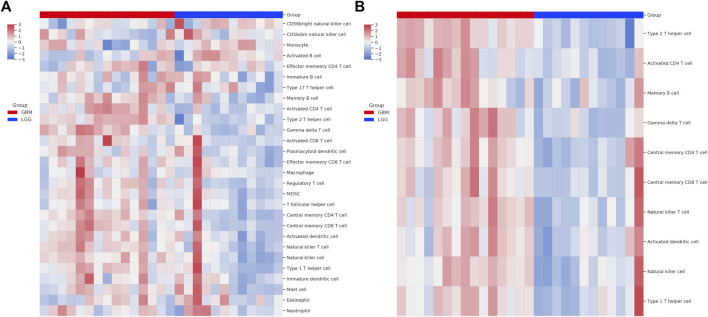
Single-sample GSEA (ssGSEA) analysis of immune cell infiltration in tumor. **(A)** Heatmap of 28 immune related cell types in patients; **(B)** heatmap of the types of immune cells passed the significant level in Student’s t test.

We next used the GSEA enrichment analysis to evaluate the differences in KEGG cataloged pathways between grade II/III and GBM patients. We defined gene sets with *p*-value <0.05 and FDR q value < 0.25 as statistically significant. Strikingly, almost all the significant differentially expressed pathways were upregulated in the GBM group (Figure 2A). The most prominent pathways (ranked according to the NES value) are related to DNA replication and genome integrity maintenance, including pathways of mismatch repair, cell cycle, nucleotide excision repair, homologous recombination, and DNA replication. To further understand the effect of chromosome instability, we performed the positional analysis of GSEA. The results support a defect in DNA replication and chromosomal stability in the GBM group. Chromosomal bands 10q24, 10q26, and 10q22 were significantly enriched in grade II/III patients (Figure 2B). Moreover, our results also showed that chr12q12, chr7p14, and chr1p31 were enriched in GBM, although have no significance (Figure 2B). From the RNA-seq analysis, we also ran ssGSEA and focused on 28 types of infiltrating immune cells in the tumor microenvironment (Figure 3A). Grade II/III and GBM patients showed distinct immune cell infiltration patterns. Of the 28 types of infiltrating immune cells, GBM patients exhibited enhanced overall infiltration compared to grade II/III patients. We further screened significant events by using a *t*-test and found that most of the infiltrating immune cell types upregulated in GBM were related to immune activation, including CD4 T cells, CD8 T cells, dendritic cells, natural killer cells, and Type 1 T helper cells, although the repressive regulatory T cells were upregulated in the same group of patients as well (Figure 3B).

### Somatic Mutation Landscape of Grade II/III and GBM Patients

**FIGURE 4:**
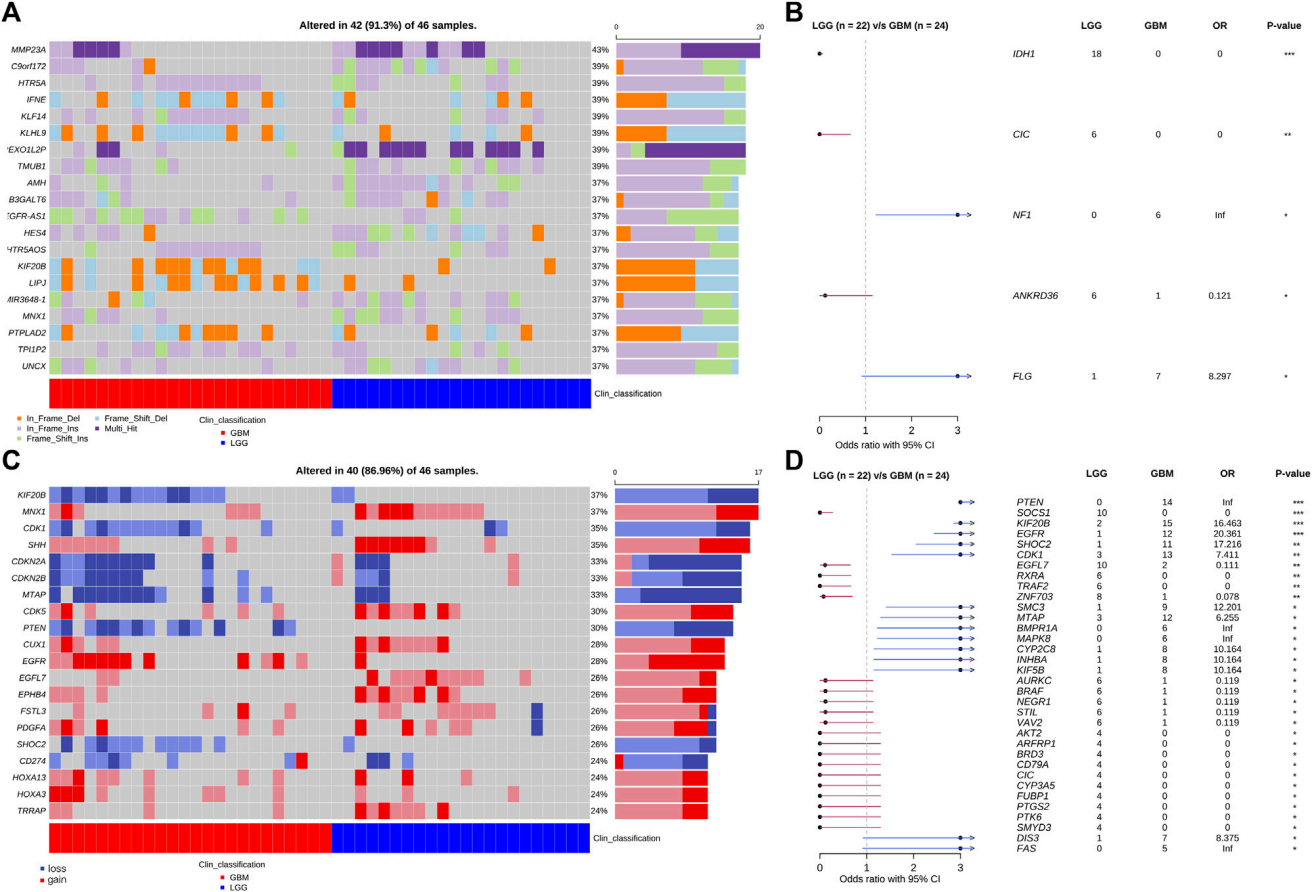
Somatic SNV, indel, and CVN mutations of patients obtained from WES analysis. **(A)** Top 20 genes with the highest SNV/Indel mutation frequency in the patient cohort. **(B)** Forest plot of SNV genes with significant distribution difference in LGG and GBM patients. **(C)** Top 20 genes with the highest CNV frequency in the patient cohort. **(D)** Forest plot of CNV genes with significant distribution difference in LGG and GBM patients. *: *p* < 0.05; **: *p* < 0.01; ***: *p* < 0.001.

The grade II/III group included 17 grade II and 5 grade III patients. The GBM group included 24 patients. We performed the WES analysis on their tumor samples and identified somatic mutations including single nucleotide variation (SNV), short insertion and deletion (indel), and copy number variation (CNV) (Figure 4). Among the genes with the highest mutation rates in the cohort, we identified four, *IDH1*, *CIC*, *NF1*, ANKRD36, and *FLG*, that have statistically different distributions between the LGG and GBM groups (Figures 4A,B). *IDH1*, *CIC*, and *NF1* have been repetitively reported in gliomas and the preferential distribution of *IDH1* in the LGG group verified the clinical classification of our patient cohort. Additionally, 94% (18/19) detected *IDH1* mutations were R132H, and there was only one R132S substitution (data not shown). There were more genes with CNV that had significantly different frequencies between the grade II/III and GBM groups (Figures 4C,D). Several genes achieve a significance level of *p* < 0.01 in Fisher’s exact test (Figure 4D). These genes include *PTEN*, *SOCS1*, *KIF20B*, *EGFR*, *SHOC2,* and *CDK1*. Almost all the CNV genes, except *SOCS1* and *EGFL7*, are enriched in the LGG group. Interestingly, *PTEN*, *KIF20B*, *SHOC2,* and *EGFL7* had copy number loss in all the affected patients and *EGFR* is the only one that had copy number gain in all the affected patients.

### Somatic Alterations Related to Prognosis

**FIGURE 5:**
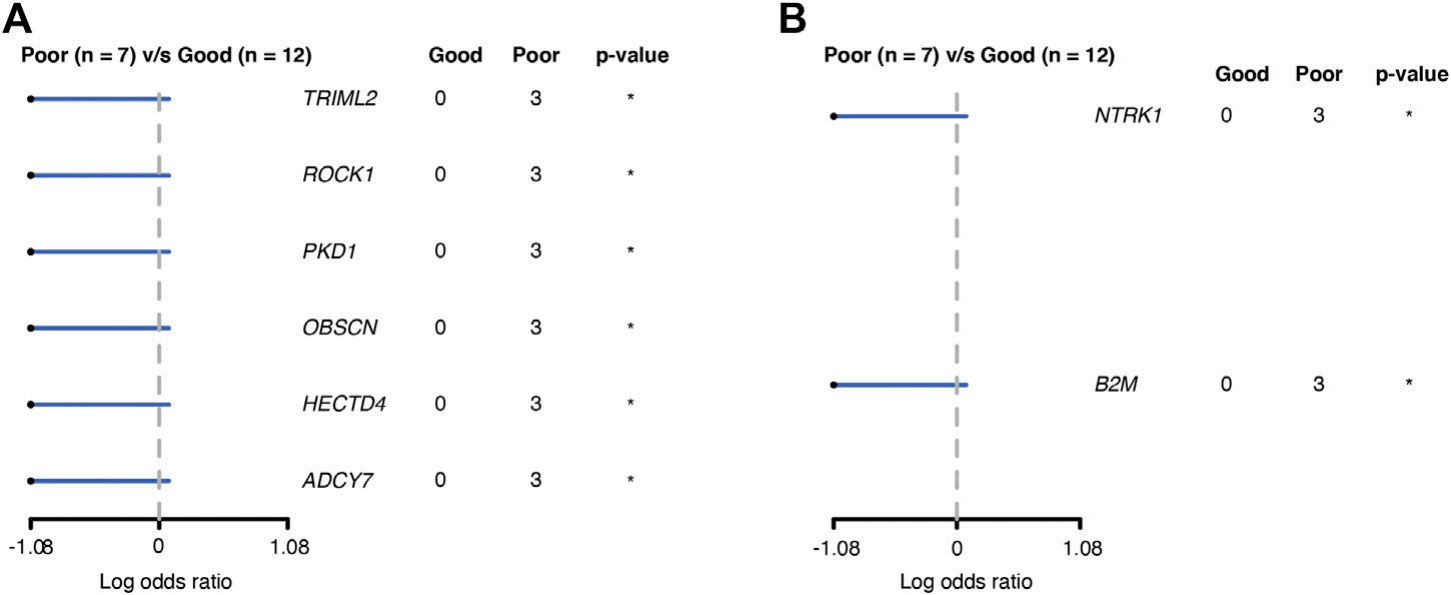
Genes associated with PFS6 prognosis in GBM patients. **(A)** Genes with SNV; **(B)** genes with CNV. *: *p* < 0.05.

GBM is known for its poor prognosis but there is still a wide range of response times to treatment among GBM patients. We are interested in finding potential prognostic factors among GBM patients and tried to collect the treatment response of the patients in the cohort. Nineteen of the GBM patients passed a rigorous review of their treatment and pathological exam history and were included in the final analysis. Considering the poor prognosis of GBM in general, we set six-month progression-free survival (PFS6) after surgery as the endpoint for treatment response. Seven GBM patients recurred within 6 months and were classified as the poor PFS6 group. The other 12 GBM patients did not have progression 6 months past surgery and were classified as the good PFS6 group. Cross-tabulation analysis showed that there were no significant differences in baseline clinical characteristics that have contributed to prognosis between the good and poor groups, including age, gender, treatment, and tumor location (Supplementary Table S1). Comparing the somatic mutations between the two groups, we found six genes (*TRIML2*, *ROCK1*, *PKD1*, *OBSCN*, *HECTD4,* and *ADCY7*) that had significantly higher mutation frequency (Figure 5A) and two genes (*NTRK1* and *B2M*) had higher CNV frequency in the poor group (Figure 5B). All these somatic variants were exclusively presented in the poor group, which might be potential indicators of poor survival in GBM.

## Discussion

In his study, we empowered WES with RNA-seq data to characterize the molecular and gene expression difference of grade II/III and GBM patients in the Chinese population, and also compared the GMB patients with different PFS6 statuses. In this study, we provided valuable evidence regarding the chromosome instability and immune-hot phenotype of GBM, and novel variant genes with low frequencies that may enable the stratification of GBM patients with good or worse prognoses.

According to the WHO classification, grade I and grade II are labeled as low-grade glioma (LGG) depending on their histopathological features (Louis et al., 2016; Louis et al., 2021). However, in the Cancer Genome Atlas (TCGA) database, LGG was defined to consist of grade II and III gliomas (Gittleman et al., 2020). Both types of definitions have their strengths but emphasized a different aspect of similarity or dissimilarity of grade I, II, and III gliomas. For the WHO definition, grade III is considered of high malignancy and is placed in the same group as GBM. Even though grade III gliomas are indeed malignant, they still are not as aggressive as GBM. From an unsupervised clustering analysis of the gene expression profiles of our patient cohort, the result supports grade II and III gliomas, which share a large degree of similarity (Figure 1). We, therefore, adopted the TCGA convention and based our comparative analysis on the grouping between LGG and GBM. In the previous study, Teo et al. illustrated the three subtypes predominance among the populations of Caucasians, Koreans, and Chinese according to the RNA profiling of each patient, as well as showed the different responses by the radiochemotherapies between the subtype groups (Teo et al., 2019). Therefore, this may be the reason that the four GBM patients were clustered as LGG in our study cohort.

In our cohort, the genomic profiles of LGG and GBM resemble those from the available public database such as TCGA, PanCancer Altas, and cBioPortal. However, we do find several differences between our cohort, which is based on the Chinese patient population, and the public databases, which are overwhelmingly Western/European populations. In addition to some commonly mutated genes such as *IDH1*, *EGFR*, *PTEN*, and *CIC*, we discover several novel mutant genes with different distributions between the LGG and GBM groups. The mutation frequencies of these genes are higher than that of the TCGA population. For example, the CNV of KIF20B accounts for 56.5% in the GBM and 8.7% in the LGG group of our cohort. However, in the TCGA-GBM cohort, it only appears in 1% of patients. Other genes such as *SMC3*, *CYP2C8*, and *SHOC2* show a similar pattern, that is, a high appearance frequency in our cohort and differentially in the GBM group, but a much lower overall frequency in the TCGA-GBM group. This deviation between our cohort and the TCGA cohort is unlikely due to the difference between sequencing and mutant calling procedure. As supporting evidence, we detected *EGFR* amplification frequencies of 45% in GBM and 8% in LGG. These frequencies are consistent with the TCGA-GBM cohort. It is possible that the difference in patient ethnic backgrounds contributed to the difference in the CNV frequencies in the aforementioned genes (*PTEN*, *SOCS1*, *KIF20B*, *RGFR*, and *SHOC2*). However, we need to point out that the patient number in our cohort is relatively small, which can be a factor in the uncertainty of gene mutation frequency counting.

Through comparison of the transcriptome data between the LGG and GBM groups, we found that several cellular pathways are differentially enriched in GBM. Most of these pathways are related to DNA replication and DNA damage repair functions. On the other hand, we observed a significant loss of chromosome 10 bands in the GBM patients through the WES analysis which is consistent with the early reports in GBM (del Mar Inda et al., 2003). Combining the above two pieces of results, we believe genome instability is a high-frequency event in GBM, which may contribute to the tumor malignancy in GBM. We detected PTEN CNV loss in 50% of GBM patients and also significant deletion of chromosomal band 10q23, where PTEN is located. Functional studies have shown that PTEN played an essential role in maintaining chromosomal integrity and loss of PTEN could affect homologous recombination and induce alterations of chromosome (Shen et al., 2007; Yin and Shen, 2008). These reports are in-line with our observations and further support a genome instability phenotype in GBM patients.

We also used RNA-seq data to explore immune cell infiltration in the tumor microenvironment. We found that the GBM patients exhibited an immune-hot phenotype with enhanced immune cell infiltration compared with LGG patients, a result also reported by recent studies (Feng et al., 2020). Our result showed that the activated CD8 T cells and central memory CD8 T cells were significantly enriched in HGG compared with LGG, yet no difference was seen in T-cell exhaustion between the two groups (Figure 3). This in theory should lead to a good efficacy of immunotherapy. However, the efficacy of immunotherapy in GBM in reality is very limited in clinical treatment. Factors other than immune cell infiltration, for example, tumor accessibility of immune inhibitors and the effectiveness of the infiltrated immune cells, could be limiting factors of treatment efficacy of the current regiments under testing in gliomas. More carefully selected patients and a more sophisticate design of treatment protocol is needed to improve the efficacy of immunotherapy in gliomas.

At present, the standard treatment for GBM still follows the landmark Stupp protocol in which patients were first operated then treated by radiotherapy and/or temozolomide-based chemotherapy. The protocol has given a meaningful survival advantage to GBM patients compared to treatment otherwise, but the majority of patients, nevertheless, would progress and had a PFS for less than 1 year. In our search for prognostic factors in GBM patients, we used PFS6 to divide GBM patients into good and poor prognosis groups. Genome-wide screening with WES data found that alteration of several genes was associated with poor prognosis. Among these genes, ROCK1 and B2M have been reported to be functionally related to the progress of GBM (Zhou et al., 2016; Louca et al., 2020; Zhang et al., 2021) and raise the possibility that they may be potential prognostic factors. However, the small patient number in our study prevented us to draw a conclusion.

## Conclusion

Taken together, our genomic and transcriptome analysis revealed that GBM showed distinct characteristics in several dimensions including individual gene, chromosomal stability, and immune cell infiltration as compared to LGG. Our study also revealed several Chinese population-specific genetic changes associated with GBM and potential prognostic factors in this group of patients.

## Data Availability

Publicly available datasets were analyzed in this study. This data can be found here: European Genome-Phenome Archive, EGAS00001005583.
