# Supplementary material for: Integrative Genomic and Transcriptomic Analysis of Primary Malignant Gliomas Revealed Different Patterns Between Grades and Somatic Mutations Related to Glioblastoma Prognosis
Source: Front Mol Biosci. 2022 Jul 5;9:873042. doi: 10.3389/fmolb.2022.873042 (PMC9294235; doi:10.3389/fmolb.2022.873042)
Supplement: Supplementary file 1 [file Table1.docx]

**Table S1** **Characteristics of patients in “poor” and “good” groups**

| **Characteristic** | **All**  **(n = 19)** | **Poor**  **(n = 7)** | **Good**  **(n = 12)** | **p-value** |
| --- | --- | --- | --- | --- |
| **Gender** |  |  |  |  |
| Female | 9 | 4 | 5 | 0.515 |
| Male | 10 | 3 | 7 |  |
| **Age, year** |  |  |  |  |
| < 60 | 14 | 5 | 9 | 0.865 |
| ≥ 60 | 5 | 2 | 3 |  |
| **Treatment** |  |  |  |  |
| Chemotherapy | 15 | 5 | 10 | 0.539 |
| Chemoradiotherapy | 4 | 2 | 2 |  |
| **MGMT Methylation** |  |  |  |  |
| Yes | 12 | 4 | 8 | 0.678 |
| No | 7 | 3 | 4 |  |
| **Location** |  |  |  |  |
| Temporal | 11 | 3 | 8 | 0.611 |
| Frontal  Other | 5  3 | 2  2 | 3  1 |  |
